# Fluorinated Amphiphilic Dendrimer to Improve PET Imaging of Cancer

**DOI:** 10.1002/smll.202511972

**Published:** 2026-05-05

**Authors:** Béatrice Louis, Tom Roussel, Twiany Cruz‐Dubois, Ling Ding, Zhenbin Lyu, Erik Laurini, Jessica Ou, Laure Balasse, Adrien Chabert, Vincent Nail, Ahlem Bouhlel, Françoise Dignat‐George, Florence Gattacceca, Sabrina Pricl, Benjamin Guillet, Ling Peng, Philippe Garrigue

**Affiliations:** ^1^ INSERM, INRAE, C2VN Aix Marseille University Marseille France; ^2^ CNRS, CERIMED Aix Marseille University Marseille France; ^3^ Aix Marseille University, CNRS, Centre Interdisciplinaire de Nanoscience de Marseille (CINaM) Equipe Labellisée Ligue Contre le Cancer Marseille France; ^4^ Molecular Biology and Nanotechnology Laboratory, Department of Engineering and Architectures University of Trieste Trieste Italy; ^5^ INSERM, CNRS, INRIA, CRCM Aix Marseille University Marseille France; ^6^ Pôle Pharmacie, Radiopharmacie Assistance Publique Hôpitaux de Marseille Marseille France; ^7^ Department of General Biophysics, Faculty of Biology and Environmental Protection University of Lodz Lodz Poland

**Keywords:** amphiphilic dendrimer, cancer, fluorination, imaging, PET, self‐assembling, tumor

## Abstract

Early and accurate tumor detection is key to successful cancer management. Nanotechnology‐based biomedical imaging offers strong potential to enhance tumor detection through improved sensitivity and specificity, particularly via the enhanced permeability and retention (EPR) effect. However, suboptimal biodistribution of imaging agents is often associated with a pronounced liver retention. Fluorinated compounds, though rare in nature, provide unique physicochemical properties thanks to the high electronegativity of fluorine, its strong inductive effect, and the low polarizability of the C‐F bond, conferring exceptional chemical stability and biological inertness. Here, we report a fluorinated supramolecular dendrimer nanosystem for tumor imaging using positron emission tomography (PET). This system was constructed via the self‐assembly of an amphiphilic fluorinated dendrimer radiolabeled with gallium‐68, a positron‐emitting radionuclide commonly used for PET. This fluorinated radiotracer exhibited a nanoscale size and had high radiochemical purity and stability. Compared to its non‐fluorinated analogue, the fluorinated system showed reduced liver accumulation, improved pharmacokinetics, and significantly enhanced tumor uptake in orthotopic and ectopic mouse xenograft models of glioblastoma and pancreatic adenocarcinoma. These results demonstrate that fluorination is a powerful strategy to optimize pharmacokinetics and biodistribution of imaging agents, enabling more accurate and effective tumor detection.

## Graphical abstract

Fluorination of a self‐assembling dendrimer nanosystem reduced liver retention, accelerated renal clearance and improved biodistribution, ultimately enhancing tumor uptake and enabling more sensitive tumor detection by positron emission tomography (PET). This fluorination strategy offers a promising avenue for the rational design and optimization of nanosystems dedicated to cancer imaging.

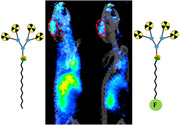

## 1 Introduction

Bioimaging plays a crucial role in cancer management for accurate diagnosis and treatment. Among imaging modalities, positron emission tomography (PET) stands out for its high sensitivity and quantitative capability, serving as a powerful tool for tumor diagnosis to assess staging, grading, as well as monitoring treatment response and antitumor efficacy [1, 2, 3, 4]. However, further improvements in targeting specificity and spatial resolution are still in high demand. Nanotechnology‐based imaging offers a promising route to address these challenges and improve imaging sensitivity and specificity by exploiting the enhanced permeability and retention (EPR) effect alongside the multiplied number of radionuclides per nanoparticle. The EPR effect is a characteristic of solid tumors and their microenvironments related to their anatomical and pathophysiological differences from normal tissues, enabling nanoparticles to enter the tumor tissue and accumulate there owing to the leaky vasculatures and the disabled lymphatic system [5]. As a result, the local concentration of imaging agents in tumor lesions can be significantly increased, leading to better imaging sensitivity and specificity.

**FIGURE 1:**
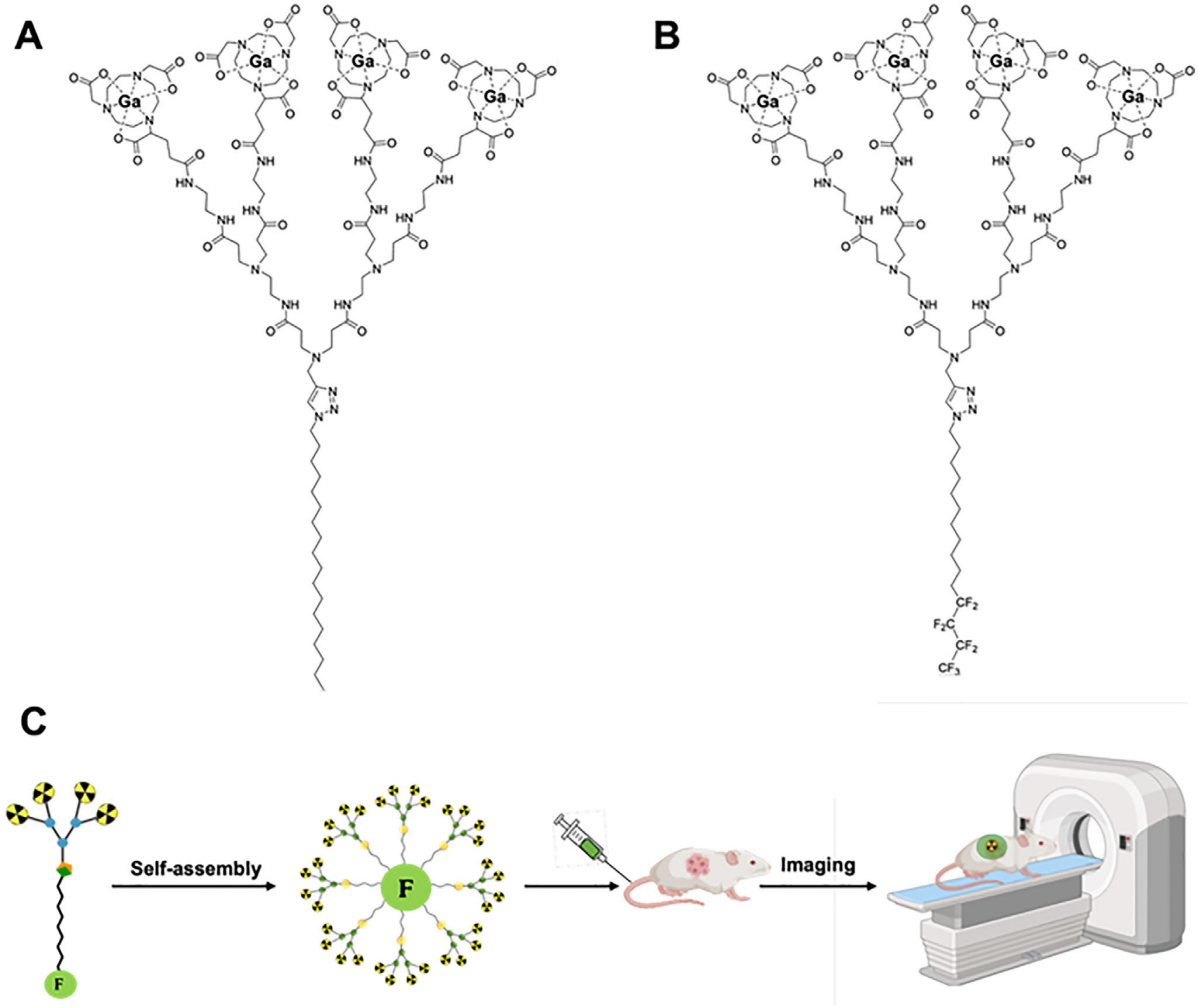
Chemical structures of the amphiphilic dendrimers (**A**) [^68^Ga]Ga‐**1** and (**B**) [^68^Ga]Ga‐**F1**, and (**C**) cartoon illustration of the self‐assembling dendrimer nanoystem for PET imaging of tumor.

Dendrimer nanosystems are particularly attractive for applications in biomedical imaging thanks to their well‐defined structure, multivalency and tunable functionality [6, 7, 8]. We have previously developed self‐assembling supramolecular dendrimer platforms for tumor imaging using positron emission tomography [9], single photon emission computed tomography (SPECT) [10] and magnetic resonance imaging [11, 12]. These dendrimer nanosystems were constructed via self‐assembly of amphiphilic dendrimers composed of a long hydrophobic alkyl chain and a small hydrophilic poly(amidoamine) (PAMAM) dendron functionalized with imaging units. Specifically, amphiphilic dendrimers terminated with macrocyclic chelators were radiolabeled with [^68^Ga]Ga^3+^ radionuclide for PET ([^68^Ga]Ga‐**1** in Figure 1A). Our further studies demonstrated that nanoparticle surface charge significantly influences biodistribution, with slightly positive surface charge exhibiting reduced liver uptake compared to those negatively charged [13]. More recently, we showed that modifying the hydrophobic domain—while maintaining the same hydrophilic dendron part—can dramatically alter biodistribution, leading to massive accumulation in the liver [14]. These findings highlight the critical role of internal molecular composition.

In this study, we investigate fluorination as a strategy to modulate biodistribution of the supramolecular dendrimer nanosystem. Fluorination is known to impart distinctive properties to organic molecules or materials, largely due to the highest electronegativity of fluorine and the unique characteristics of the C‐F bond including strong inductive effects and preferential fluorine–fluorine interactions that can stabilize supramolecular assemblies [15, 16, 17, 18, 19, 20]. Specifically, we designed a fluorinated amphiphilic dendrimer by introducing fluorine atoms into the hydrophobic alkyl chain ([^68^Ga]Ga‐**F1** in Figure 1B). We present here the development and evaluation of this novel fluorinated nanosystem as a PET imaging agent (Figure 1C), demonstrating that fluorination is an effective strategy to optimize nanotracers for improving biodistribution and tumor imaging performance.

## 2 Materials and Methods

### 2.1 Starting Materials

Dendrimer **1** was synthesized according to the well‐established protocol published in our group [9]. NODA‐GA(tBu)_3_ was purchased from CheMatech (Dijon, France). Dialysis tubing was purchased from Sigma Aldrich (St. Quentin Fallavier, France). Radiolabeling quality control analyses were performed on instant thin layer chromatography (iTLC, Agilent Technologies, Santa Clara, USA) with a MiniGITA radiochromatography system (Elisia‐Raytest, Angleur, Belgium).

### 2.2 Nuclear Magnetic Resonance

Nuclear Magnetic Resonance (NMR) experiments were acquired at 300K using a Bruker Avance DRX 500 NMR spectrometer (Karlsruhe, Germany) operating at 500 MHz for ^1^H NMR, 470 MHz for ^19^F NMR and 126 MHz for ^13^C NMR.

### 2.3 Mass Spectrometry

High resolution mass spectrometry experiments were performed with a Synapt G2 HDMS quadrupole/time‐of‐flight (Manchester, UK) equipped with an electrospray source operating in positive or negative mode when necessary.

### 2.4 Synthesis of the Amphiphilic Dendrimer 1

The synthesis and characterization of dendrimer **1** was carried out as described in our previous study [9].

### 2.5 Synthesis of the Amphiphilic Dendrimer F0

To a solution of the azido‐bearing fluorinated chain (29 mg, 0.070 mmol) and the alkynyl‐terminated PAMAM dendron (42 mg, 0.066 mmol) in THF (6.0 mL) under argon, was added CuSO_4_·5H_2_O (1.0 mg, 0.0035 mmol) in H_2_O (1.0 mL) and sodium ascorbate (1.5 mg, 0.0070 mmol) in H_2_O (1.0 mL). Then the mixture was stirred at 50°C for 3 h under argon in dark until the reaction was completed as indicated by thin layer chromatography (TLC) analysis. Then solvent was removed under reduced pressure, the obtained residue was dissolved in 20 mL CH_2_Cl_2_ and extracted with saturated 0.10 M ethylenediaminetetraacetic acid (EDTA) solution (10 mL×2), saturated NaHCO_3_ solution (10 mL×2) and brine (10 mL). The organic phase was dried with anhydrous Na_2_SO_4_, filtered and concentrated. The crude product was purified by column chromatography on silica gel with CH_2_Cl_2_/MeOH, giving the corresponding fluorinated amphiphilic PAMAM dendrimer with ester terminals as a colorless oil (56 mg, 81%).

To a solution of the above obtained dendrimer (30 mg, 0.029 mmol) in methanol (2.0 mL) under argon in ice bath, ethylenediamine (EDA) was added (0.60 mL, 8.7 mmol) slowly. The reaction mixture was stirred at 30°C for 3.0 days until the reaction was completed as indicated by IR analysis. Then the solvent was evaporated under reduced pressure, the obtained residue was purified using dialysis (dialysis tubing, MWCO 1000, changing dialysis water every hour for 6 times) and lyophilization. Repeating the operation cycles of dialysis and lyophilization 3 times gave dendrimer **F0** as a white solid (33 mg, yield: 99%). ^1^H NMR (400 MHz, CD_3_OD) δ 7.91 (s, 1H), 4.39 (t, *J* = 7.1 Hz, 2H), 3.81 (s, 2H), 3.25 (m, 12H), 2.83–2.69 (m, 20H), 2.57 (t, *J* = 6.8 Hz, 4H), 2.43 (t, *J* = 6.8 Hz, 4H), 2.36 (t, *J* = 6.8 Hz, 8H), 2.14 (tt, *J* = 19.0, 8.1 Hz, 2H), 1.90 (p, *J* = 7.2 Hz, 2H), 1.59 (p, *J* = 7.5 Hz, 2H), 1.46–1.26 (m, 14H). ^19^F NMR (376 MHz, CD_3_OD) δ ‐82.55 (br, 3F), ‐115.51 (br, 2F), ‐124.41 (br, 2F), ‐127.16 (br, 2F). HRMS‐ESI(+): calcd. *m/z* for C_48_H_90_F_9_N_16_O_6_ [M+3H]^3+^ 385.9023, found 385.9031 (+ 0.8 ppm).

### 2.6 Synthesis of the Amphiphilic Dendrimer F1

To a solution of NODA‐GA(*t*Bu)_3_ (125 mg, 0.23 mmol) in 3.0 mL anhydrous dimethylformamide (DMF) were added benzotriazol‐1‐yloxytripyrrolidinophosphonium hexfluorophosphate (PyBOP, 120 mg, 0.23 mmol) and N‐methylmorpholine (NMM, 32 µL, 0.29 mmol). The mixture was stirred for 15 min and then a solution of **F0** (22 mg, 0.019 mmol) in anhydrous DMF (1.5 mL) was added, and the resulting solution was stirred at 30°C for 3.0 days under argon. Followed by the addition of 20 mL ethyl acetate, the resulting mixture was washed with saturated NaHCO_3_ solution (3 × 4.0 mL). Then the organic layer was dried over anhydrous MgSO_4_, filtered and evaporated under reduced pressure. The obtained residue was purified by precipitation with 5.0 mL DCM/pentane v/v 0.1/10 three times and then dissolved in a TFA/CH_2_Cl_2_ mixture (3.0 mL, v/v = 1/1) and stirred at 30°C for 24 h under argon. After evaporating the solvent, the crude residue was purified by dialysis (dialysis tubing, MWCO 2000) and lyophilization. Repeating the operation cycle of dialysis (change dialysis water every hour for 8 h) and lyophilization for 4 times, the final dendrimer **F1** was obtained as a white solid (38 mg, yield: 77%). ^1^H NMR (500 MHz, CD_3_OD/D_2_O): δ 8.27 (s, 1H), 4.43 (s, 2H), 3.74‐3.65 (m, 22H), 3.50‐3.16 (m, 84H), 3.05‐2.70 (m, 30H), 2.49‐2.31 (br, 8H), 2,11‐1.85 (m, 14H), 1.52 (br, 2H), 1.37‐1.17 (br, 14H). ^19^F NMR (470 MHz, CD_3_OD/ D_2_O): δ ‐88.09 (br, 3F), ‐115.05 (br, 2F), ‐125.20 (br, 2F), ‐126.84 (br, 2F). ^13^C NMR (126 MHz, D_2_O): δ177.48, 176.30, 174.37, 173.66, 177.63, 137.50, 128.17, 66.80, 58.33, 53.23, 52.31, 51.60, 50.86, 50.51, 50.32, 50.02, 47.51, 39.99, 39.55, 35.48, 33.73, 31.25, 30.83, 30.59, 30.11, 29.94, 29.82, 29.66, 29.59, 28.39, 27.10, 26.22, 20.79. ESI(‐) HRMS: calculated isotopic maximum at *m/z* 1291.1439 for C_108_H_177_F_9_N_28_O_34_^2−^, found at *m/z* 1291.1429 (+0.8 ppm).

### 2.7 Synthesis of the Amphiphilic Dendrimer F1 Labeled with Non‐Radioactive Gallium‐69

The dendrimer **F1** (4.9 mg, 1.9 µmol) was dissolved in 2.0 mL H_2_O. To this solution was added 1.0 mL solution of [^69^Ga]GaCl_3_ (1.4 mg, 8.3 µmol) in 1.0 mM HCl and the pH value was adjusted to 4.5 with addition of 0.20 m ammonium acetate. The mixture was stirred for 15 min at 25°C. The obtained crude product was purified by dialysis (dialysis tubing, MWCO 2000) to yield the corresponding [^69^Ga]Ga**‐F1** solution. ESI(+) HRMS: calculated isotopic maximum at *m/z* 951.3105 for C_108_H_170_F_9_Ga_4_N_28_O_34_^3+^ found at *m/z* 951.3102 (‐0.3 ppm).

### 2.8 Transmission Electron Microscopy

Transmission electron microscopy (TEM) was performed using JEOL 2100F analytical electron microscope (Tokyo, Japan) to characterize the size and morphology of the nanoparticles at an accelerating voltage of 200 kV. The dendrimer was dispersed in milliQ water at a concentration of 1.0 mg/mL, and vortex for 30 s, then diluted to 1.6 µg/mL, followed by depositing an aliquot (4.0 µL) onto a carbon‐coated copper grid and dried at 25°C for 15 min. The grid was then stained with 3.0 µL uranyl acetate (2.0% in aqueous solution) for 5 s, and the excess uranyl acetate was removed by filter paper before measurements.

### 2.9 Dynamic Light Scattering Analysis

Dynamic light scattering (DLS) analysis was performed to determine the size and size distribution of the nanoparticles. Specifically, the dendrimer was first dispersed in milliQ water at a concentration of 0.50 mg/mL, and vortexed 30 s, then the fresh solution was measured using a Zetasizer Nano ZS (Malvern, United Kingdom) equipped with a standard 633 nm LASER at 25°C. Measurements were achieved with disposable solvent resistant micro cuvette (ZEN0040) for size and size distribution, and with disposable folded capillary zeta cell (DTS1070) for zeta potential. All experiments were done in triplicates.

### 2.10 Critical Micelle Concentration

Critical micelle concentration (CMC) was determined using fluorescence spectroscopy with Nile Red as the fluorescence probe. [^69^Ga]Ga‐**F1** dendrimer solutions at different concentrations varying from 1.0 × 10^−7^ to 2.0 × 10^−4^ mol/L were prepared, with the final Nile Red concentration of 3.0 × 10^−6^ mol/L in water. The solutions were vortexed for 10 min and kept for 2 h at room temperature to promote the micelle formation prior to fluorescence measurement. Fluorescence spectra were recorded at the emission wavelength of 635 nm on F‐4500 fluorescence spectrophotometer (CARY Eclipse) at room temperature. Excitation wavelength is 550 nm. The normalized fluorescence intensity was analyzed as a function of dendrimer concentration.

### 2.11 Isothermal Titration Calorimetry

Isothermal titration calorimetry (ITC) experiments were performed with a MicroCal PEAQ‐ITC calorimeter (Malvern, UK) at 37°C in HEPES buffer pH = 6.5. The cell volume was 208 µL. The thermodynamic parameters for the complexation of Ga^3+^ and **F1** were investigated in buffered solutions. Specifically, a solution of 200 µM of **F1** (at concentration well above their CMC) was titrated with 18 step‐by‐step injections of 2.0 µL volume of 8.0 mM GaCl_3_ (in syringe). For the determination of the CMC value, 2.0 µL of a [^69^Ga]Ga‐**F1** dendrimer buffered solution in HEPES, at an optimized concentration of 1200 µM, was injected 18 times at 150 s intervals into the calorimeter sample cell filled with a solution of HEPES buffer. All solutions and buffer were degassed for 30 min at room temperature under stirring at 600 rpm prior to each experiment. After careful washing, the cell was pre‐rinsed with a portion of the buffer solutions. Upon filling cell and syringe, stirring was turned on and the system was allowed to thermally equilibrate for 30 min.

### 2.12 Radiolabeling of Dendrimers with Gallium‐68

Freshly prepared 1.0 mol/L ammonium acetate solution (0.060 mL) was added to 0.050 mL dendrimer solution of **1** or **F1** (50 µg/50 µL in milliQ water). To this solution was added 0.50 mL of a [^68^Ga]GaCl_3_ solution freshly eluted from a pharmaceutical grade ^68^Germanium/^68^Gallium generator (Galliapharm, Eckert & Ziegler Berlin, Germany), using 0.10 mol/L HCl. The solution was incubated for 10 min at room temperature. The resulting solution at pH = 5.0 was then neutralized with 10 µL NaOH 0.10 mol/L to reach pH 7.0 and vortexed. No centrifugation or purification step was required. The complex was obtained with a radiochemical purity superior to 95%, as previously performed [9]. This radiochemical purity was determined using iTLC (solid phase: iTLC‐SG (Agilent, Les Ulis, France); mobile phase: 0.10 mol/L sodium citrate pH = 5.0). The solution was diluted to appropriate concentrations and then used for in vitro and in vivo studies without further purification.

### 2.13 Radiolabeling Stability

The radiolabeling stability of [^68^Ga]Ga**‐1**@ and [^68^Ga]Ga**‐F1**@ was assessed by incubating 0.10 mL of the radiotracer solution right after their radiolabeling, in 0.40 mL of human serum and in 0.40 mL of NaCl 0.9%, respectively. The stability of all radiolabeled dendrimers was checked at both 25°C and 37°C for up to 4 h in NaCl 0.9% and in human serum. The radiochemical purity stability was checked 0 h, 1, 2, 3, and 4 h after the radiolabeling, using iTLC with a miniGITA radiochromatograph (Elysia Raytest, Angleur, Belgium).

### 2.14 Octanol/Water Partition Coefficient Measurement

The lipophilicity of gallium‐68 radiolabeled nanotracers was evaluated using the octanol/water partition method. Briefly, 100 µL of [^6^ ^8^Ga]Ga‐**F1**@ or [^68^Ga]Ga‐**1**@ was mixed with 400 µL of distilled water, followed by the addition of 500 µL of octanol. The biphasic system was vortexed vigorously for 3 min and centrifuged at 6000 rpm for 5 min to achieve phase separation. Aliquots (100 µL) of each phase were collected separately, and the radioactivity was measured using a γ‐counter. The log P value was calculated as the logarithm of the ratio of radioactivity in the octanol phase to that in the aqueous phase. The log P values were of ‐0.58 ± 0.47 for [^68^Ga]Ga‐**1**@ and ‐1.0 ± 0.1 for [^6^ ^8^Ga]‐**F1**@, respectively.

### 2.15 Animal Care and Use

All procedures using animals were approved by the Institutional Animal Care and Use Committee (C2EA‐71, INT, Aix‐Marseille University) and by the French Ministry of Higher Education and Research (project #32157, #31843 and #14177) and conducted according to the EU Directive 2010/63/EU and the ARRIVE 2.0 guidelines [21]. Animals were housed in enriched cages placed in a temperature‐ and hygrometry‐controlled room with daily monitoring, fed with water and commercial diet *ad libitum*. The choice of mouse strain for each tumor model followed prior validation and established protocols for these models, to ensure reproducibility and comply with the Reduction principle (3R) by avoiding additional optimization studies, to ensure robust engraftment and growth kinetics for each model.

### 2.16 Healthy Mice Biodistribution

Nine‐week‐old healthy Swiss male mice (Charles River, *n* = 24) were injected (100 µL) in tail vein with 4.1 ± 0.76 MBq of [^68^Ga]Ga‐**1**@ or 3.7 ± 0.50 MBq of [^68^Ga]Ga‐**F1**@ with twelve mice per group under isoflurane anesthesia (2.0%) for two hours. Six mice of each group were injected on the camera, and a dynamic PET/CT acquisition was launched for two hours. In [^68^Ga]Ga‐**F1** group one mouse was excluded due to extravasation during injection. Six other mice of each group were dedicated to blood sampling analysis.

Dynamic small animal PET/CT imaging was performed using a NanoScan PET/CT scanner (Mediso, Budapest, Hungary). The PET acquisition parameters included 4 iterations, a coincidence window of 1–3, and a 10 cm field of view (FOV). The CT scan was conducted over the same FOV as the PET, with the following parameters: 35 kV voltage, 300 ms exposure, 480 projections, and a semi‐circular acquisition method. Attenuation‐corrected CT reconstruction was carried out using Nucline software (v.3.04.025.0000, Mediso, Budapest, Hungary). PET data were reconstructed across the following time frames: 0–5 min, 6–10 min, 11–15 min, 16–20 min, 21–25 min, 26–30 min, 31–45 min, 46–60 min, 61–75 min, 76–90 min, 91–105 min, and 106–120 min. Quantitative volumes of interest (VOIs) were determined on attenuation‐ and decay‐corrected PET/CT images using VivoQuant software (v.3.52022 patch1, Invicro, USA). CT‐based VOIs were manually defined to assess and compare the accumulation of dendrimer formulations in specific organs including the brain, heart, liver, lungs, kidneys, bladder and muscle, based on PET signal quantification. The results were expressed as the percentage of the injected dose per gram of tissue (%ID/cm^3^).

The blood sampling was collected at 5, 15, 30, 45, 60, 75, 90, 105, and 120 min post‐injection and analyzed using gamma‐counting (Wizard 2480, Perkin‐Elmer) after decay correction. For both groups at two hours post‐injection, mice were euthanized, and organs (kidneys, liver, heart, lungs, intestines, spleen, pancreas, bone, muscle and brain) were collected, washed in PBS (Eurobio‐scientific, Les Ulis, France), weighed and analyzed by gamma‐counting. Results were decay‐corrected and expressed as %ID/g.

### 2.17 Xenograft Model of Pancreatic Cancer

Human pancreatic adenocarcinoma xenografts were established by orthotopic injections of 1 × 10^6^ SOJ‐6 cells (primary pancreatic adenocarcinoma, 0.025 mL Roswell Park Memorial Institute (RPMI) 1640 culture media (ThermoFisher Scientific, Waltham, USA) with 10% fetal calf serum and 0.025 mL Matrigel Matrix (Corning, New York, USA)) in NMRI nude mice (Janvier Labs, *n* = 5). Each mouse was injected with 1 × 10^6^ SOJ‐6 cells/50 µL into the pancreas. Animals were allowed to rest for 3 weeks. Five SOJ‐6 tumor‐bearing mice were injected with a contrast agent for CT (ExiTron nano 12000, 100 µL, retro‐orbital injection, Miltenyi Biotec, Bergisch Gladbach, Germany) one day before the injection of the radiotracer. The mice were injected in the tail vein with 3.8 ± 0.31 MBq of [^68^Ga]Ga‐**1**@ and with 4.8 ± 0.4 MBq of [^68^Ga]Ga‐**F1**@ under isoflurane anesthesia (2%), maintained for 2 h until a 20min‐long PET/CT static acquisition was launched. Another PET/CT static acquisition was carried out 4 h post‐injection to assess late biodistribution.

### 2.18 Xenograft Model of Glioblastoma

Ectopic human glioblastoma xenografts were obtained by subcutaneous injection in the black flank of 5.0 × 10^6^ U87 cells (human glioblastoma) in 0.050 mL of PBS (Lonza, Basel, Switzerland) with 10% fetal calf serum, after trypsinization (0.05% Trypsin‐EDTA, Lonza), to athymic nude mice (Charles River, Wilmington, MA, USA, *n* = 6 per group). Animals were then allowed for resting for three weeks. These mice were injected in the tail vein with 5.0 ± 0.24 MBq of [^68^Ga]Ga‐**1**@ and with 5.5±0.7 MBq of [^68^Ga]Ga‐**F1**@ under isoflurane anesthesia (2%). A PET/CT static acquisition was launched 2 and 4 h post injection.

Orthotopic human glioblastoma xenografts were also established by stereotaxic injection of 0.5 × 10^6^ U87 cells (3.0 µL, in PBS (Mg^2^^+^/Ca^2^^+^) into 8‐week‐old female athymic nude mice (Janvier Labs, *n* = 8) under 2% isoflurane anesthesia. Stereotaxic injections using a Hamilton microsyringe were realized in the left striatum (coordinates:—2 mm dorsal/ventral relative to the dura mater, + 1 mm lateral, and + 1 mm anterior/posterior from bregma). Three weeks later, these mice were injected in the tail vein with 5.4 ± 1.1 MBq of [^68^Ga]Ga‐**1**@ and with 5.2 ± 0.53 MBq of [^68^Ga]Ga‐**F1**@ under isoflurane anesthesia (2 %). A PET/CT static acquisition was done 4 h post injection.

### 2.19 PET/CT Acquisitions and Image Treatment

Static small animal PET/CT imaging was performed over a 20‐min acquisition using a NanoScan PET/CT scanner (Mediso, Budapest, Hungary). The PET acquisition parameters included 4 iterations, a coincidence window of 1–3, and a 10 cm field of view (FOV). The CT scan was conducted over the same FOV as the PET, with the following parameters: mean duration 6 min, 35 kV voltage, 300 ms exposure, 480 projections, and a semi‐circular acquisition method. Attenuation‐corrected CT reconstruction was carried out using Nucline software (v.3.04.025.0000, Mediso, Budapest, Hungary). Quantitative volumes of interest (VOIs) were determined on attenuation‐ and decay‐corrected PET/CT images using VivoQuant software (v.3.52022 patch1, Invicro, USA). CT‐based VOIs were manually defined to assess and compare the accumulation of dendrimer formulations in specific organs including the brain, heart, liver, lungs, kidneys, bladder and muscle, based on PET signal quantification. The results were expressed as the percentage of the injected dose per gram of tissue (%ID/cm^3^).

### 2.20 Non‐compartmental Pharmacokinetic Analysis

Non‐compartmental analysis (NCA) was achieved using PKanalix software v2023 (Lixoft, Antony, France). NCA was used to describe organ exposure via the area under the plasma concentration‐time curve extrapolated to infinity (AUC_inf_) and the main pharmacokinetic parameters in blood for each formulation: clearance, half‐life, and volume of distribution.

### 2.21 Statistical Analysis

All statistical analyses were performed with Prism v9.0 (GraphPad, San Diego, CA, USA). Data were presented as mean values ± SD. Normality of data distribution was verified with the Shapiro‐Wilk test. Data were submitted either to a one‐way ANOVA followed by a Dunn‐Šidák's post‐hoc test when comparing more than two groups with a single factor, or a two‐way ANOVA followed by a Dunn‐Šidák's post‐hoc test when comparing more than two groups with two factors. Data were submitted to an unpaired t‐test for direct comparisons between two groups. *P* < 0.05 indicated statistical significance.

## 3 Results

### 3.1 Reliable Synthesis of the Fluorinated Dendrimer and Chelation with Ga^3^^+^

**FIGURE 2:**
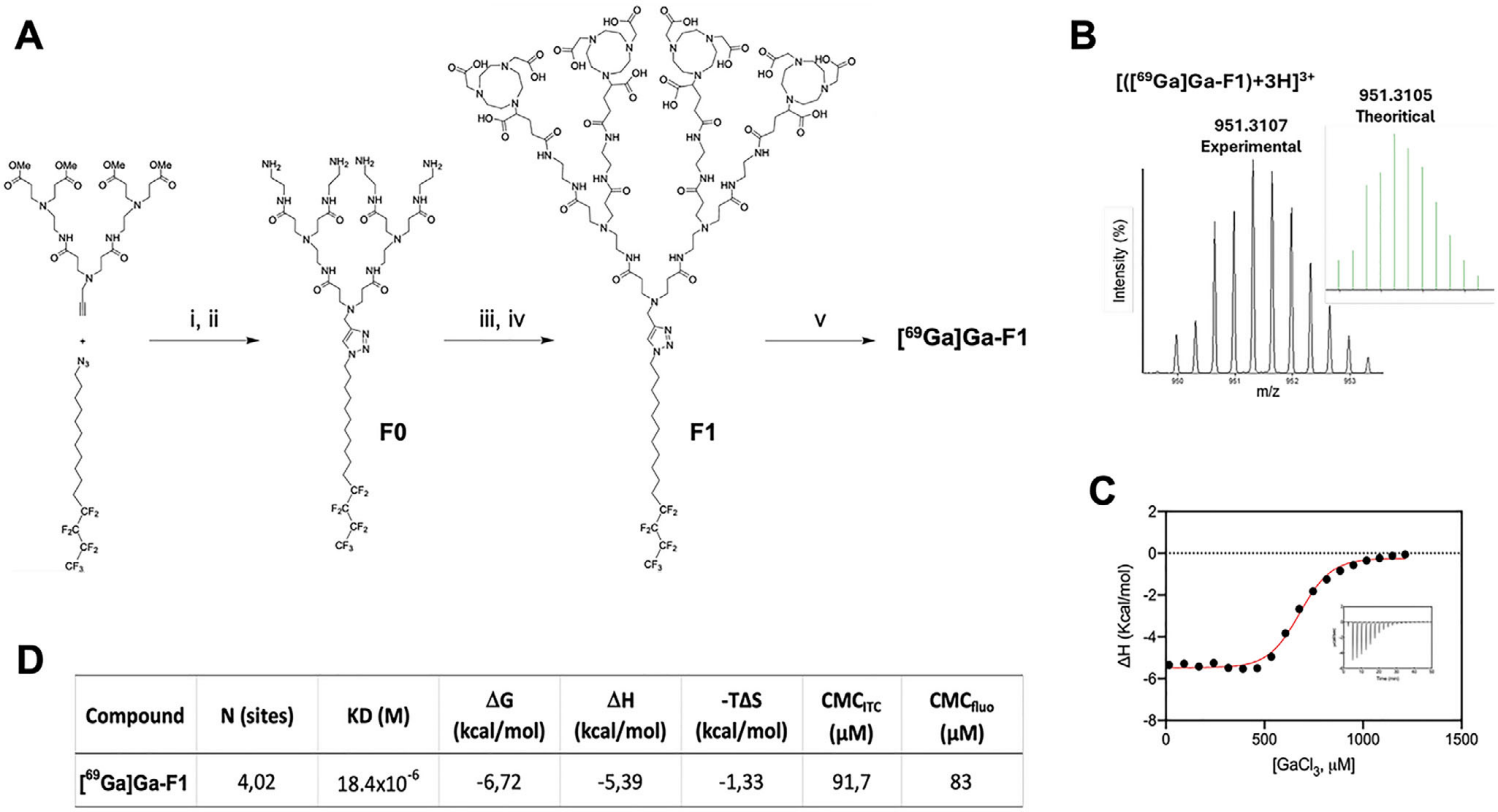
(A) Chemical synthesis of the fluorinated dendrimer F1 and its chelation complexes [^69^Ga]Ga‐F1 and [^68^Ga]Ga‐F1 with the nonradioactive isotope [^69^Ga]Ga^3+^ and the radioactive isotope [^68^Ga]Ga^3+^, respectively. (i) CuSO_4_, Sodium Ascorbate, THF/H_2_0, 25°C, 15 min (ii) Ethylenediamine, MeOH, 30°C, 72 h; (iii) NODA‐GA(tBu)_3_, PyBOP, NMM, DMF, 30°C, 72 h; (iv)TFA, CH_2_Cl_2_, 30°C, 16 h. (v) [^69^Ga]GaCl_3_, 1.0 m HCl, 0.20 m ammonium acetate, 25°C, 15 min. (B) High‐resolution mass spectrum showing the isotopic pattern characteristic of the triply charged species [[^69^Ga]Ga‐F1+3H]^3+^. The inset shows the calculated isotopic pattern. (C) Isothermal titration calorimetry (ITC) curve for chelation of Ga^3+^ with dendrimer F1. The inset shows measured heat power vs. time elapsed during titration. (D) Summary table of the thermodynamic parameters, N number of occupied NOTA macrocycle, ΔG Gibbs energy, ΔH enthalpy, ΔS entropy, CMC_ITC_ and CMC_fluo_ are the critical micellar concentration obtained respectively from isothermal titration calorimetry and Nile red method.

The synthesis of the fluorinated amphiphilic dendrimer F1 (Figure 2A) started with the dendrimer precursor F0, which was prepared by coupling the azido‐bearing fluorinated chain with the alkynyl‐terminated PAMAM dendron via the copper‐catalyzed click chemistry (Figure 2A; Scheme S1). Similar to the synthesis of dendrimer 1 [9], F0 was conjugated with the reagent NODA‐GA(tBu)_3_, followed by deprotection to yield the fluorinated dendrimer F1 (Figure 2A). The chemical structure and integrity of F1 were analyzed using ^1^H, ^13^C, and ^19^F NMR and high‐resolution mass spectrometry (HRMS). Specifically, ^19^F NMR spectrum revealed the characteristic signals corresponding to the fluorinated dendrimer: four ^19^F NMR peaks were assigned to the corresponding fluorinated entity attached on the carbon chain (Figure S2). Moreover, the characteristic signals of the NOTA cages were apparent on the ^1^H and ^13^C NMR. Further HRMS confirmed the molecular mass and integrity: the experimental mass/charge obtained for the doubly charged species matched the theoretical molecular weight within an error of less than 0.8 ppm.

Chelation of **F1** with the stable isotope ^69^Ga^3+^ by using ^69^GaCl_3_, followed by dialysis to remove free ^69^Ga^3+^, provided the non‐radioactive dendrimer [^69^Ga]Ga‐**F1** (Figure 2A). HRMS showed the characteristic isotopic pattern and the exact molecular weight of the triply charged species [^69^Ga]Ga‐**F1**+3H]^3+^ similar to the theoretical prevision (Figure 2B). To support the reliable synthesis of [^69^Ga]Ga‐**F1**, the formation of the complex between dendrimer **F1** and ^69^Ga^3+^ was further investigated using isothermal titration calorimetry (ITC) (Figure 2C). The binding capacity between Ga^3+^ and **F1** demonstrated the spontaneous formation of the complex (ΔG = −6.72 kcal/mol), resulting from the balanced and favorable contributions of both the enthalpic (ΔH = −5.39 kcal/mol) and the entropic component (−TΔS = −1.33 kcal/mol) (Figure 2D). Finally, the molar ratios identified by the ITC‐derived number of occupied sites 4.02 (N sites in Figure 2D) was close to the theoretical stoichiometry of 4:1 for the [^69^Ga]Ga‐**F1** complex.

### 3.2 [^69^Ga]Ga‐F1 Self‐Assembled into Small, Uniform and Stable Nanoparticles

**FIGURE 3:**
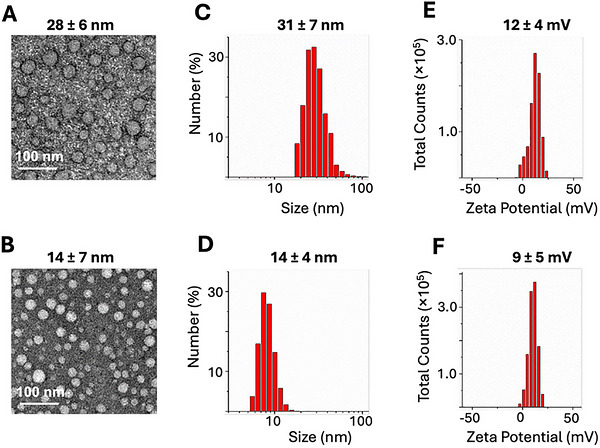
Self‐assembly of the amphiphilic dendrimers [^69^Ga]Ga‐F1 and [^69^Ga]Ga‐1 in aqueous solution forming small and uniform nanomicelles [^69^Ga]Ga‐F1@ (upper row) and [^69^Ga]Ga‐1 (lower row). (A,B) Transmission electron microscopic micrograph, (C,D) size and size distribution and (E,F) surface zeta‐potential of [^69^Ga]Ga‐F1@ and [^69^Ga]Ga‐1, respectively.

Owing to the intrinsic amphiphilicity, [^69^Ga]Ga‐**F1** self‐assembled spontaneously in aqueous solution. Results of transmission electron microscopy (TEM) revealed that [^69^Ga]Ga‐**F1** self‐assembled into small, uniform and spherical nanomicelles (denoted as [^69^Ga]Ga‐**F1**@ thereafter) with average diameter around 28 nm (Figure 3A). The effective formation of nanomicelles in solution was further confirmed using dynamic light scattering (DLS) analysis, highlighting an average size of 31 nm (Figure 3C) which was coherent with the TEM analysis. We further determined the critical micelle concentration (CMC) of [^69^Ga]Ga‐**F1** using a well‐reported method operating fluorescence spectroscopic assay with Nile Red, and the CMC of [^69^Ga]Ga‐**F1** was around 80 µM (Figure S3). This value was in line with that obtained using ITC (92 µM) (Figure 2C,D).

It is to note that the surface zeta potential of [^69^Ga]Ga‐**F1**@ was slight positive (+12 mV) (Figure 2E), which is similar to that of the [^69^Ga]Ga‐**1** nanomicelles (denoted as [^69^Ga]Ga‐**1**@ thereafter): + 9 mV (Figure 3F).

### 3.3 Effective Radiolabeling with High Radiolabeling Purity and Stability

**FIGURE 4:**
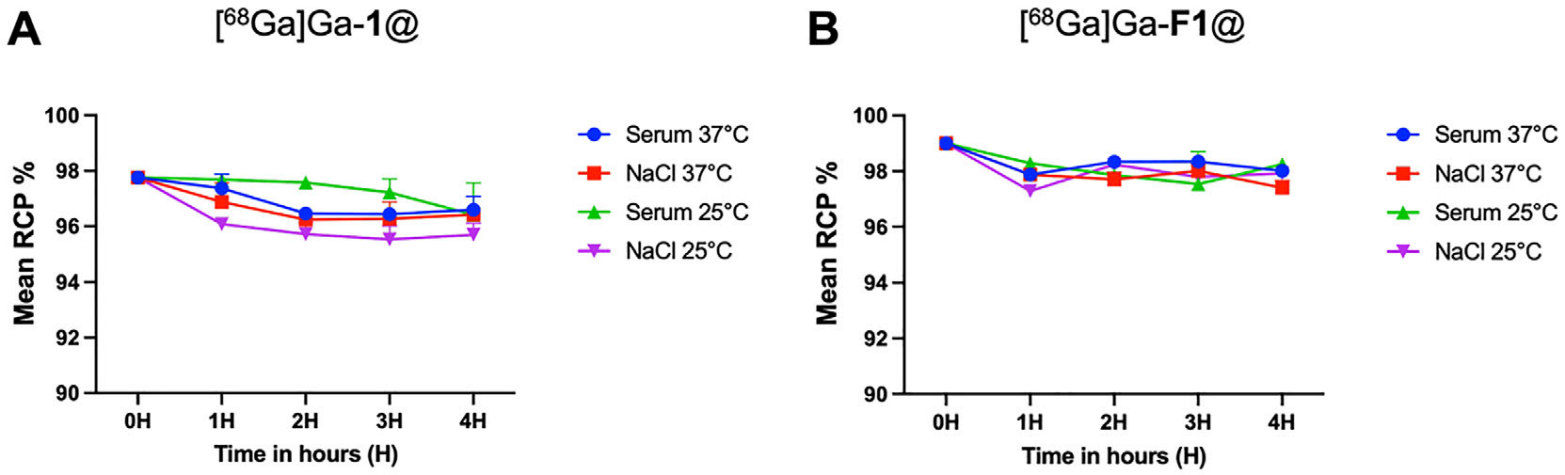
Radiochemical purities (RCP) and stabilities in physiological serum and in human serum for 4 h at 25°C and at 37°C for (**A**) [^68^Ga]Ga‐**1**@ and (**B**) [^68^Ga]Ga‐**F1**@. Data were submitted to a two‐way ANOVA, with no significant difference over time or over incubation conditions.

Further labeling of **1** and **F1** with radioactive [^68^Ga]GaCl_3_ yielded [^68^Ga]Ga‐**1**@ and [^68^Ga]Ga‐**F1**@, respectively. Both [^68^Ga]Ga‐**1**@ and [^68^Ga]Ga‐**F1**@ were obtained with a radiochemical purity higher than 95% and remained stable in physiological serum and in human serum at least for 4 h at 25°C and at 37°C (Figure 4; Tables S1 and S2). Interestingly, fluorination resulted in a slight decrease in lipophilicity of [^68^Ga]Ga‐**F1**@ (log P = ‐1.0 ± 0.1) compared to that of [^68^Ga]Ga‐**1**@ (log P  = ‐0.58 ± 0.47). This is not surprising as fluorination often alters both hydrophobicity and lipophobicity.

### 3.4 Fluorination Resulted in a More Favorable Biodistribution in Healthy Animals

**FIGURE 5:**
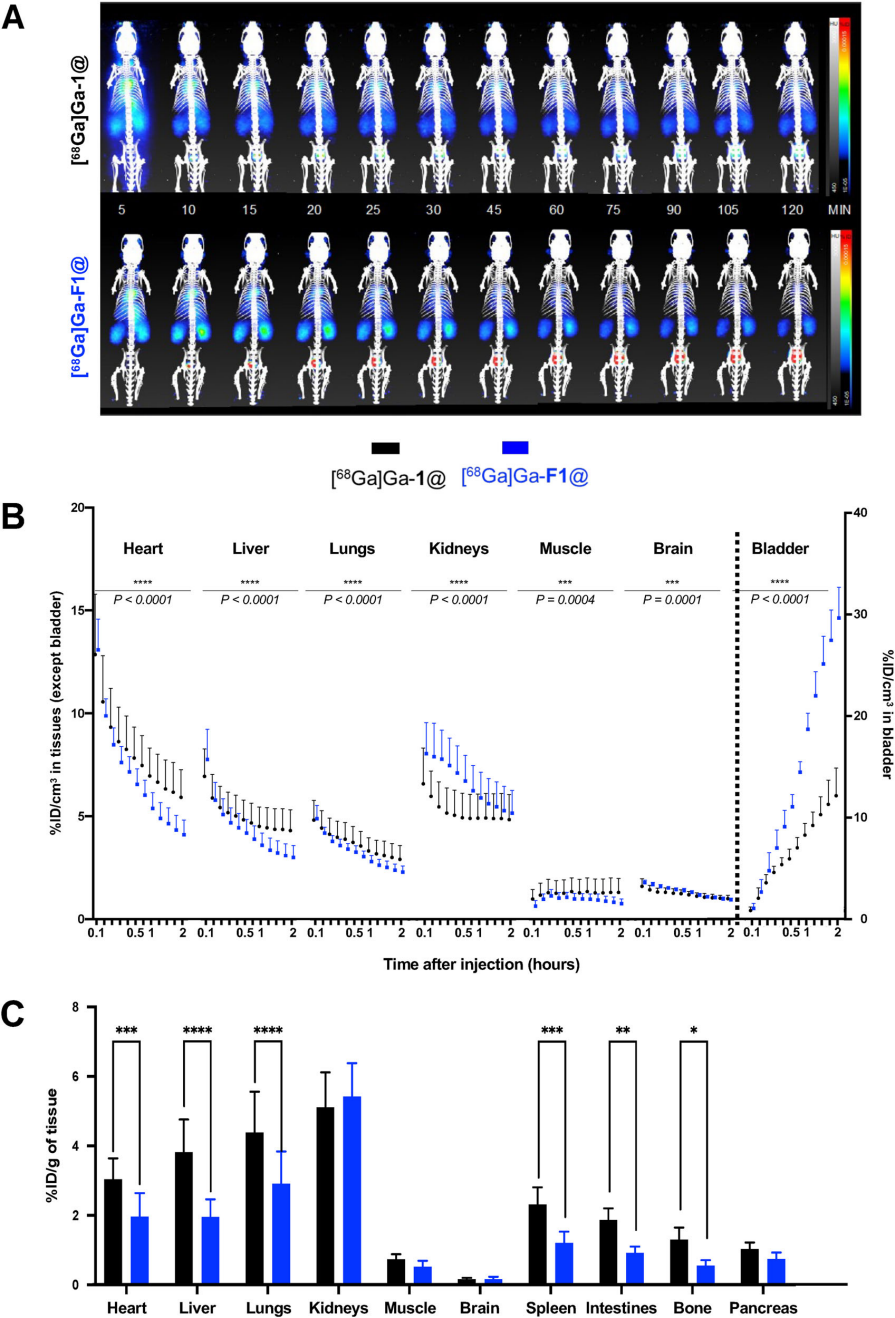
PET/CT biodistribution study up to 2 h after injection in healthy mice with [^68^Ga]Ga‐1@ (*n* = 6) or [^68^Ga]Ga‐F1@ (*n* = 5). (A) Representative maximum intensity projection PET/CT images and (B) associated PET signal quantifications. For statistical analysis by two‐way ANOVA for (A) and (B): ^*^ *p* <0.05, ^**^ *p* <0.01, ^***^ *p* <0.001, ^****^ *p* <0.0001. (C) Ex vivo gamma‐counting of main organs 2 h after injection with either [^68^Ga]Ga‐1@ (*n* = 12) or [^68^Ga]Ga‐F1@ (*n* = 11). For statistical analysis by one‐way ANOVA for (C): ^*^ *p* <0.05, ^**^ *p* <0.01, ^***^ *p* <0.001, ^****^ *p* <0.0001.

In vivo biodistribution was first studied in healthy animals, through PET imaging and gamma‐counting (Figure 5). [^68^Ga]Ga‐F1@ PET signal was quantified and compared with that of [^68^Ga]Ga‐1@, revealing a significant decrease in the liver (respectively 3.00 ± 0.58 %ID/cm^3^ and 4.31 ± 1.01 %ID/cm^3^, ^****^ *p* <0.0001), in the heart (respectively 4.11 ± 0.71 %ID/cm^3^ and 5.92 ± 1.35 %ID/cm^3^, ^****^ *p* <0.0001), in the lungs (respectively 2.29 ± 0.30 %ID/cm^3^ and 2.91 ± 0.67 %ID/cm^3^, ^****^ *p* <0.0001), in the muscle (respectively 0.76 ± 0.23 %ID/cm^3^ and 1.30 ± 0.68 %ID/cm^3^, ^***^ *p* = 0.0004) and in the brain (respectively 0.95 ± 0.07 %ID/cm^3^ and 1.00 ± 0.16 %ID/cm^3^, ^***^ *p* = 0.0001) at 120 min post‐injection. Oppositely, an increased [^68^Ga]Ga‐F1@ PET signal was quantified in the bladder compared with [^68^Ga]Ga‐1@ (respectively 27.45 ± 2.99 %ID/cm^3^ and 12.15 ± 2.71 %ID/cm^3 ****^ *p* <0.0001) and in the kidneys (respectively 5.15 ± 1.11 %ID/cm^3^ and 4.84 ± 1.21 %ID/cm^3^) (Figure 5A,B).

Gamma‐counting confirmed the decreased PET signal quantifications of [^68^Ga]Ga‐F1@ compared with [^68^Ga]Ga‐1@ in the liver (respectively 1.95 ± 0.51 %ID/g and 3.82 ± 0.94 %ID/g, ^****^ *p* <0.0001), in the spleen (respectively 1.20 ± 0.33 %ID/g and 2.30 ± 0.50 %ID/g, ^***^ *p* = 0.0001), in the intestines (respectively 0.92 ± 0.18 %ID/g and 1.90 ± 0.33 %ID/g, ^**^ *p* = 0.0016), in the heart (respectively 1.96 ± 0.68 %ID/g and 3.04 ± 0.60 %ID/g, ^***^ *p* = 0.0002), in the lungs (respectively 2.90 ± 0.93 %ID/g and 4.40 ± 1.20 %ID/g, ^****^ *p* <0.0001) and in the bone (respectively 0.55 ± 0.16 %ID/g and 1.30 ± 0.19 %ID/g, ^*^ *p* = 0.0264). No significant difference was found in the kidneys (respectively 5.42 ± 0.96 %ID/g and 5.11 ± 1.01 %ID/g) (Figure 5C).

### 3.5 Fluorination Improved Pharmacokinetic Parameters

**FIGURE 6:**
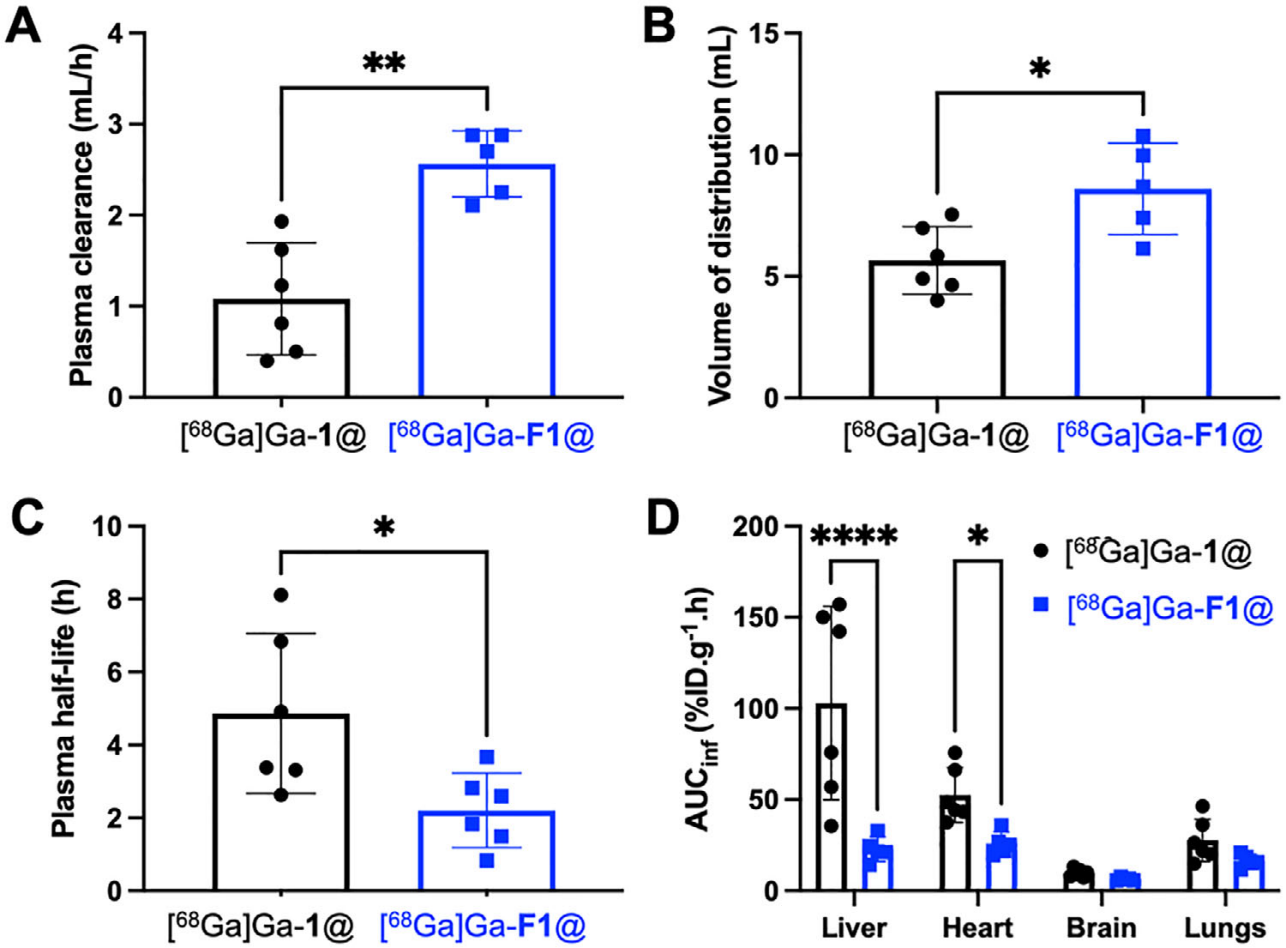
Pharmacokinetic data: (A) Plasma clearance, (B) volume of distribution, (C) plasma half‐life and (D) exposures in the liver, heart, brain and lungs (AUC_inf_) of [^68^Ga]Ga‐1@ and [^68^Ga]Ga‐F1@. Data mean ± SD (*n* = 6) were submitted to an unpaired *t*‐test (A, B and C) or a one‐way ANOVA (D) ^*^ *p* <0.05, ^**^ *p* <0.01, ^***^ *p* <0.001, ^****^ *p* <0.0001.

Non‐compartmental pharmacokinetic analysis was performed on dynamic PET quantifications and concluded that [^68^Ga]Ga‐**F1**@ displayed a significantly higher plasma clearance than [^68^Ga]Ga‐**1**@ (respectively 2.56 ± 0.36 mL/h and 1.08 ± 0.62 mL/h, ^**^ *p* = 0.0011, Figure 6A). The volume of distribution (Vd) of [^68^Ga]Ga‐**F1**@ was also significantly higher compared to that of [^68^Ga]Ga‐**1**@ (respectively 8.60 ± 1.88 mL and 5.66 ± 1.39 mL, ^*^ *p* = 0.0151, Figure 6B). A significant shorter plasma half‐life was found for [^68^Ga]Ga‐**F1**@ compared to [^68^Ga]Ga‐**1**@ (respectively 2.21 ± 1.02 h and 4.86 ± 2.19 h, ^*^ *p* = 0.0227, Figure 6C). Total exposures (AUC_inf_) to [^68^Ga]Ga‐**F1**@ in the main organs were lower than for [^68^Ga]Ga‐**1**@, especially in the liver (respectively 22.9 ± 6.8 %ID/g.h and 102.9 ± 53.1 %ID/g.h, ^****^ *p* <0.0001) and in the heart (respectively 25.9 ± 6.3%ID/g.h and 52.6 ± 14.9 %ID/g.h, ^*^ *p* = 0.0455), and to a less extent in the brain (respectively 6.4 ± 0.8 %ID/g.h and 9.8 ± 2.2 %ID/g.h, *P* = 0.7923) and in the lungs (respectively 16.4 ± 3.6 %ID/g and 27.7 ± 11.6 %ID/g, *P* = 0.3870, Figure 6D).

### 3.6 Fluorination Improves PET Imaging of Tumor Models

**FIGURE 7:**
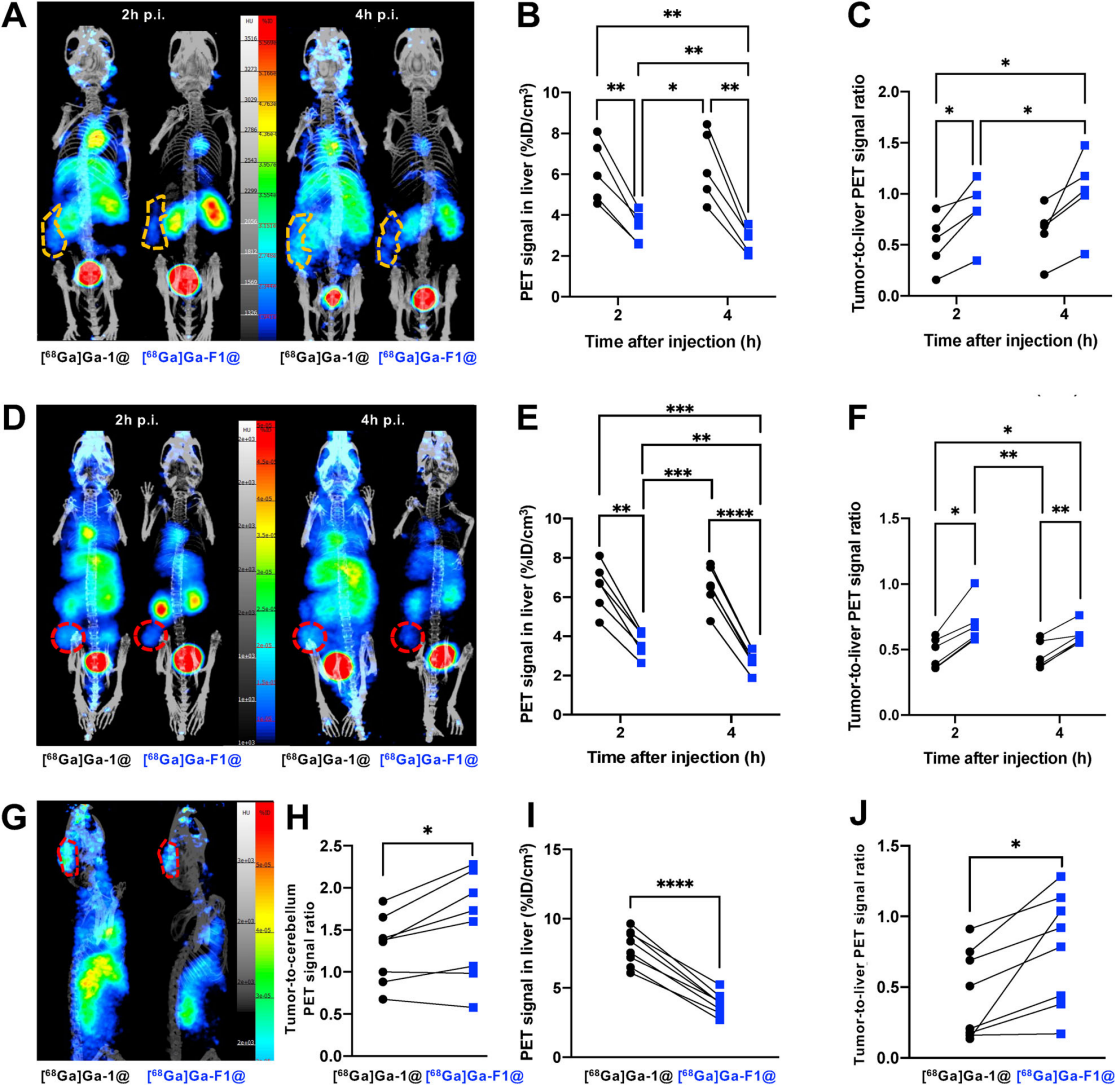
Comparison of [^68^Ga]Ga‐1@ and [^68^Ga]Ga‐F1@ for tumor imaging using PET. A) PET/CT representative images of [^68^Ga]Ga‐1@ or [^68^Ga]Ga‐F1@ 2 and 4 h post‐injection in orthotopic SOJ‐6 mouse model of pancreatic adenocarcinoma, delineated by orange dashed lines (*n* = 5). (B) Associated PET signal quantifications in the liver and (C) tumor‐to‐liver ratios. (D) PET/CT representative images of [^68^Ga]Ga‐1@ or [^68^Ga]Ga‐F1@ 2 and 4 h post‐injection in ectopic U87 mouse model of glioblastoma, delineated by red dashed lines (*n* = 5). (E) Associated PET signal quantifications in the liver and (F) tumor‐to‐liver PET signal ratios. (G) PET/CT representative images of [^68^Ga]Ga‐1@ and [^68^Ga]Ga‐F1@ at 4 h post‐injection in orthotopic U87 glioblastoma mouse model, delineated by red dashed lines (*n* = 8). (H) Associated PET signal quantifications as tumor‐to‐background (cerebellum) ratio, (I) in the liver, and (J) tumor‐to‐live PET signal ratio. Data were submitted to a two‐way ANOVA for (B), (C), (E) & (F), and to a paired t‐tested for (H), (I), and (J): ^*^ *p* <0.05, ^**^ *p* <0.01, ^***^ *p* <0.001, ^****^ *p* <0.0001.

Encouraged by the favorable biodistribution of the fluorinated nanosystem, we further conducted tumor detection using PET imaging on an orthotopic pancreatic adenocarcinoma mouse model and an ectopic glioblastoma mouse model. In both tumor‐xenograft models, [^68^Ga]Ga‐**F1**@ PET globally resulted in a lower background and better contrasted images compared to [^68^Ga]Ga‐**1**@ (Figure 7A,D). In the orthotopic pancreatic adenocarcinoma SOJ‐6 mouse model (*n* = 5), the quantified [^68^Ga]Ga‐**F1**@ PET signal in the liver was significantly decreased by 45% compared with that of [^68^Ga]Ga‐**1**@ 2 h after injection (respectively 3.4 ± 0.78 %ID/cm^3^ and 6.1 ± 1.5 %ID/cm^3^, ^**^ *p* = 0.0093) and even decreased by 56% 4 h after injection (respectively 2.8 ± 0.6 %ID/cm^3^ and 6.4 ± 1.7 %ID/cm^3^, ^**^ *p* = 0.0089) (Figure 7B). Oppositely, [^68^Ga]Ga‐**F1**@ tumor‐to‐liver PET signal ratio was significantly increased by 36% compared with that of [^68^Ga]Ga‐**1**@ 2 h after injection (respectively 0.83 ± 0.31 and 0.53 ± 0.26, ^*^ *p* = 0.0428). [^68^Ga]Ga‐**F1**@ tumor‐to‐liver PET signal ratio significantly increased by 19% 4 h after injection compared to 2 h after injection (respectively 1.02 ± 0.39 and 0.83 ± 0.31, ^*^ *p* = 0.0316) (Figure 7C).

Similarly, in the ectopic glioblastoma U87 mouse model (*n* = 5), [^68^Ga]Ga‐**F1**@ PET signal in the liver was significantly decreased by 44% compared to that of [^68^Ga]Ga‐**1**@ 2 h after injection (respectively 3.66 ± 0.59 %ID/g and 6.53 ± 0.95 %ID/cm^3^, ^***^ *p* = 0.0001) and even decreased by 57% 4 h after injection (respectively 2.83 ± 0.50 %ID/cm^3^ and 6.55 ± 0.97 %ID/cm^3^, ^****^ *p* <0.0001) (Figure 7E). [^68^Ga]Ga‐**F1**@ tumor‐to‐liver PET signal ratio was significantly increased by 32% compared with that of [^68^Ga]Ga‐**1**@ 2 h after injection (respectively 0.69 ± 0.15 and 0.47 ± 0.10, ^*^ *p* = 0.0190). Interestingly, [^68^Ga]Ga‐**F1**@ tumor‐to‐liver PET signal ratio 2 h after injection was significantly higher than that of [^68^Ga]Ga‐**1**@ 4 h after injection (respectively 0.69 ± 0.15 and 0.46 ± 0.10, ^**^ *p* = 0.018) (Figure 7F).

Remarkably, in the orthotopic glioblastoma U87 mouse model, [^68^Ga]Ga‐**F1**@ led to a significant higher tumor‐to‐cerebellum PET signal ratio compared to that of [^68^Ga]Ga‐**1**@ (respectively 1.55 ± 0.62 and 1.27 ± 0.39, ^*^ *p* = 0.0169) (Figure 7G, 7H). Furthermore, [^68^Ga]Ga‐**F1**@ PET signal in the liver was significantly reduced compared to that of [^68^Ga]Ga‐**1**@ (respectively 3.9 ± 0.77%ID/cm^3^ and 7.9 ± 1.28 %ID/cm^3^, ^****^ *p*<0.0001) (Figure 7I) resulting in a significant higher tumor‐to‐liver ratio with [^68^Ga]Ga‐**F1**@ compared to that of [^68^Ga]Ga‐**1**@ (respectively 0.77 ± 0.40 and 0.44 ± 0.31, ^*^ *p* = 0.0118) (Figure 7J).

## 4 Discussion

We previously reported the self‐assembling dendrimer‐based radiotracer [^68^Ga]Ga‐**1**@, which exploits EPR‐driven tumor accumulation and dendrimer multivalency, outperforming the clinical standard [^18^F]FDG for PET imaging in multiple preclinical models [9]. However, its non‐negligible liver uptake prompted us to introduce a fluorinated motif into the hydrophobic domain of the amphiphilic dendrimer, yielding the fluorinated dendrimer [^6^ ^8^Ga]Ga‐**F1**. This structural modification proved effective to modulate biodistribution, reduce hepatic retention, and enhance tumor‐to‐background contrast across multiple tumor models without compromising radiolabeling quality or self‐assembly behavior. To our knowledge, this is the first example to demonstrate that fluorination of amphiphilic dendrimer to generate self‐assembling supramolecular dendrimer nanosystem for improved biodistribution and tumor imaging.

Fluorine incorporation is widely used in medicinal chemistry for increasing bioactivity, enhancing metabolic stability, and improving bioavailability and pharmacokinetics of drugs and drug candidates [16, 18, 19], because fluorine has the highest electronegativity, and the C‐F bond has the strong inductive effects and exceptional stability while being bioinert [15, 20]. Fluorinated or perfluorinated motifs are also known to exhibit strong intermolecular interactions, sometimes leading to poor solubility [17]. Interestingly, in our case, fluorination did not induce solubility issues, and the fluorinated dendrimer self‐assembled spontaneously into small and uniform nanomicelles [^6^ ^8^Ga]Ga‐**F1**@. More importantly, fluorination didn't alter the quality of radiolabeling with gallium‐68: radiochemical purities exceeded 95 % and remained stable in human serum for at least 4 h at 37°C (Figure 4), confirming full compatibility with PET imaging protocols.

In vivo PET/CT imaging and ex vivo gamma counting in healthy mice demonstrated a markedly more favorable biodistribution profile for [^6^ ^8^Ga]Ga‐**F1**@ compared to [^6^ ^8^Ga]Ga‐**1**@. Liver retention fell below 2 %ID/cm^3^ at 2 h post‐injection, accompanied by enhanced early renal elimination. Ex vivo gamma‐counting confirmed that this renal acceleration did not translate into increased kidney parenchymal retention, indicating that fluorination redirected clearance without inducing nephrotoxic accumulation. A concomitant decrease in systemic retention was observed across the heart, lungs, spleen, intestines, muscle, brain and bone. Non‐compartmental analysis results corroborated these findings. [^6^ ^8^Ga]Ga‐**F1**@ exhibited significantly faster plasma clearance, larger volume of distribution, and shorter plasma half‐life compared to [^6^ ^8^Ga]Ga‐**1**@, consistent with more efficient systemic elimination. The approximately two‐fold lower AUC_liver_/AUC_blood_ ratio for [^68^Ga]Ga‐**F1**@ further supports reduced hepatic tropism rather than mere accelerated transit, and a shorter terminal half‐life in organs likely contributes to the lower total organ exposure observed. To have a better understanding of the distribution and mechanisms involved in the elimination of dendrimers, a semi‐mechanistic population PK model will be developed for the related studies in our further research plan [22].

It should be mentioned that the mechanistic basis for reduced hepatic uptake warrants careful consideration, particularly given the counterintuitive size relationship: [^6^ ^8^Ga]Ga‐**F1**@ (28 nm) is approximately twice the size of [^6^ ^8^Ga]Ga‐**1**@ (14 nm), yet displays markedly lower liver retention, contrary to the general expectation that larger particles are more readily captured by the hepatic clearance system. Since both nanosystems share the same chemical dendron entity on the surface and comparable zeta potentials (+12 and +9 mV, respectively), surface charge cannot account for the difference, and the role of the fluorinated hydrophobic core appears decisive consistent with our previous demonstration that inner chemical entity and hydrophobicity can govern hepatic fate in this platform [14]. Beyond the intrinsic properties of the C‐F bond (high electronegativity, strong inductive effect, metabolic bio‐inertness [17, 23], the reduced hepatic uptake may also reflect modulation of the protein corona at the nanoparticle surface. This hypothesis can be assessed through protein corona profiling and in vitro cell uptake assays in future works. Regarding in vivo structural integrity, direct characterization of self‐assembled nanoparticles in biological matrices remains analytically challenging for this class of systems [9, 10, 11, 12]; however, several indirect lines of evidence argue against significant in vivo degradation: radiolabeling stability exceeded 95% in human serum over 4 h; bone retention, a sensitive indicator of free [^6^ ^8^Ga]Ga^3^^+^ release [24] was significantly lower for [^6^ ^8^Ga]Ga‐**F1**@ than for [^6^ ^8^Ga]Ga‐**1**@ (0.55 ± 0.16 vs. 1.30 ± 0.19 %ID/g, *P = 0.0264); and the overall biodistribution pattern is fully consistent with intact nanoparticle trafficking rather than degradation. From a safety perspective, the reduced bone retention of [^6^ ^8^Ga]Ga‐**F1**@ compared to [^6^ ^8^Ga]Ga‐**1**@ is reassuring given known fluorine accumulation in bone upon chronic exposure [25]. At the doses administered (<0.0023 mg F/kg in mice), fluorine exposure is several orders of magnitude below EFSA daily limits [26], and no signs of toxicity or discomfort were observed throughout the study. Nevertheless, dedicated metabolic profiling as well as long‐term toxicity assay are identified as objectives for future translational research.

The improved pharmacokinetic profile translated directly into enhanced tumor imaging performance across three independent tumor models (Figure 6). In the orthotopic pancreatic adenocarcinoma model, liver uptake was reduced by 44 % at 2 h and 56 % at 4 h post‐injection, yielding a significantly higher tumor‐to‐liver ratio. Comparable reductions were observed in the ectopic glioblastoma model (>40 % at 2 h, >57 % at 4 h). Most strikingly, in the orthotopic glioblastoma mouse model, [^6^ ^8^Ga]Ga‐**F1**@ achieved a significantly higher brain tumor‐to‐cerebellum ratio compared to [^6^ ^8^Ga]Ga‐**1**@, with the cerebellum serving as an internal reference. To our knowledge, this represents the first demonstration that fluorination alone, without active targeting, can enhance brain tumor imaging following systemic administration, whereas most studies rely on active vectorization to cross the blood‐brain barrier [27, 28, 29, 30].

Collectively, the findings of reduction of major organs exposure and faster distribution of the fluorinated dendrimer nanosystem as well as optimization of tumoral targeting encourage us to prospect a theranostic approach. This dendrimer platform can be further functionalized with targeting ligands to improve tumor specificity. The next step will be to develop and evaluate the addition of a molecular targeting, complementary to the passive EPR‐targeting approach, and to consider drug loading. As a concrete proof‐of‐concept for active targeting, we are currently evaluating RGD‐functionalized derivatives of [^6^ ^8^Ga]Ga‐**F1**@, designed to add integrin‐mediated tumor targeting to the favorable passive biodistribution profile demonstrated here. The primary hypothesis under investigation is that active RGD targeting will further enhance tumor uptake while preserving the low liver retention conferred by fluorination: a combination that would be difficult to achieve with non‐fluorinated platforms given their intrinsically higher background. These results will be reported in a forthcoming publication.

## 5 Conclusion

The fluorinated dendrimer‐based PET radiotracer [^68^Ga]Ga‐**F1**@ developed in this study exhibited substantially improved in vivo performance compared to its non‐fluorinated counterpart [^68^Ga]Ga‐**1**@, including reduced liver uptake, enhanced renal clearance, and superior tumor‐to‐background contrast. This study highlights that judicious introduction of a fluorine motif into nanomaterials offers a uniquely promising avenue to tune pharmacokinetic properties and biodistribution of nanotracers for more effective and accurate tumor imaging.

## Author Contributions

PG, BG and LP coordinated the project; TR, LD and ZL synthesized the agents; TR, TCD, LD and BL performed the characterization; EL performed ITC experiments, BL, TCD, AB and VN performed the radiosyntheses; BL, TCD, PG and LB in vivo imaging acquisitions and image treatments; JO and FG performed the pharmacokinetic analyses; TR, LD, EL, BL, TCD, LB, VN, SP, PG and LP analyzed the data; TR, BL, TCD, PG, JO, FG, FDG, BG and LP wrote the paper. TR, BL, AB, EL, SP, PG and LP revised the manuscript. All authors proofed the manuscript.

## Conflicts of Interest

The authors declare no conflicts of interest.

## Supporting information




**Supporting File**: smll73594‐sup‐0001‐SuppMat.pdf.

## Data Availability

The data that support the findings of this study are available from the corresponding author upon reasonable request.
