# Supplementary material for: Fluorinated Amphiphilic Dendrimer to Improve PET Imaging of Cancer
Source: Small. 2026 May 5;22(35):e11972. doi: 10.1002/smll.202511972 (PMC13288792; doi:10.1002/smll.202511972)
Supplement: Supplementary file 1 — Supporting File: smll73594‐sup‐0001‐SuppMat.pdf. [file SMLL-22-e11972-s001.pdf]

Fluorinated amphiphilic dendrimer to improve PET imaging of cancer

Supporting Information

Table of content

Scheme S1: Synthesis of the fluorinated dendrimer precursor F0 2

Figure S1: ^{1}H, ^{19}F, NMR and HRMS of the fluorinated dendrimer F0 3

Figure S2: ^{1}H, ^{13}C, ^{19}F, NMR of the fluorinated dendrimer F1 4

Figure S3: Critical micelle concentrations 5

Table S1 : Mean radiochemical purity values for [^{68}Ga]Ga-1@ stability....6

Table S2 : Mean radiochemical purity values for [^{68}Ga]Ga-F1@ stability .6

Table S3 : Mean PET quantifications values for [^{68}Ga]Ga-1@ and [^{68}Ga]Ga-F1@ 6

Scheme S1: Synthesis of the fluorinated dendrimer precursor F0

Synthesis of hydrophobic tail

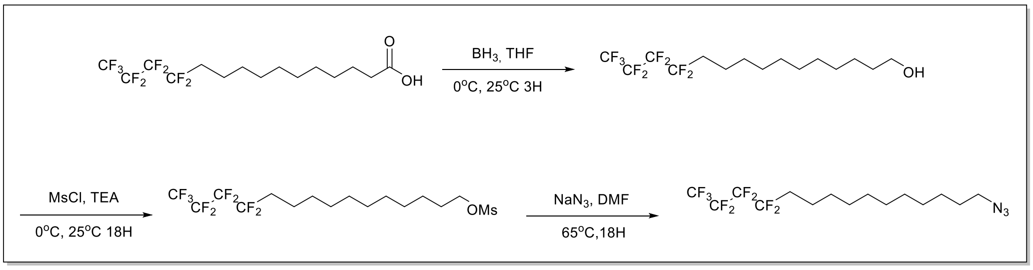

Synthesis of hydrophilic PAMAM dendron

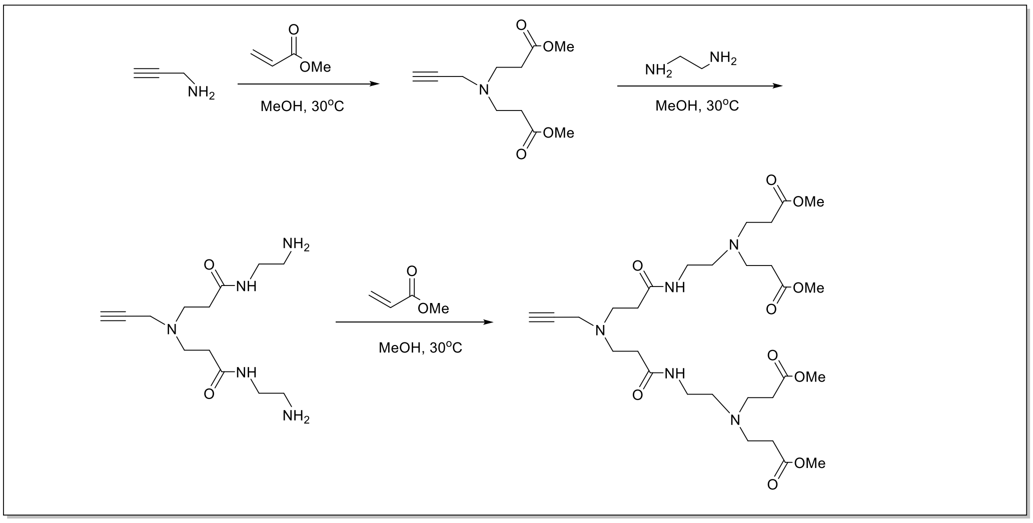

Synhtesis of amphphilic dendrimer by click chemistry

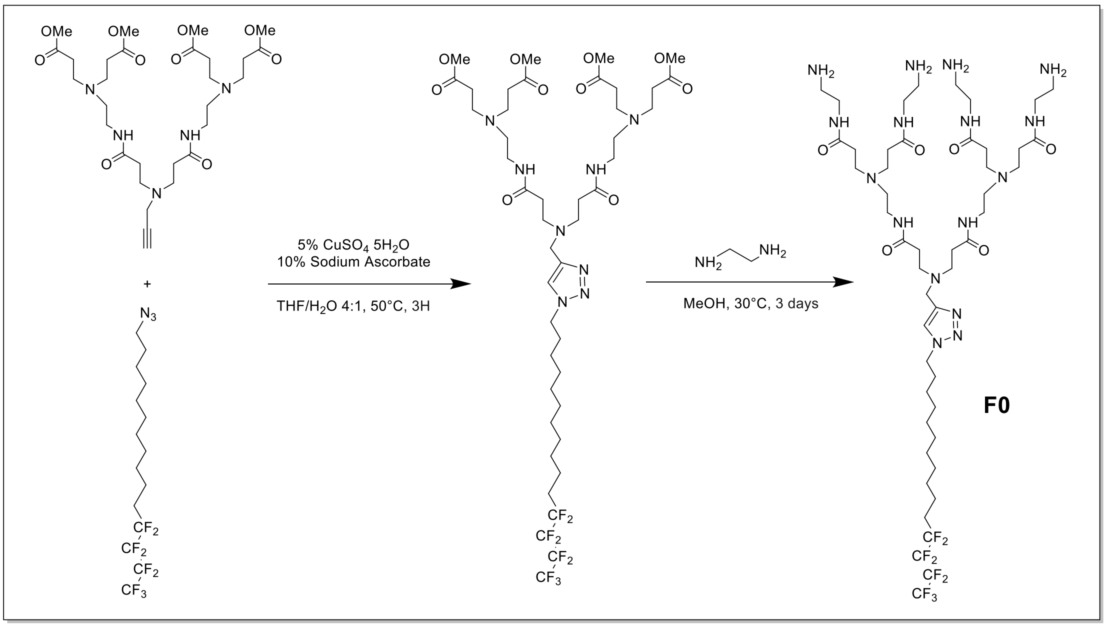

Figure S1: ^{1}H, ^{19}F, NMR and HRMS of the fluorinated dendrimer F0

(**A**) ^{1}H NMR 400 MHz (**B**) ^{19}F NMR 376 MHz spectra of the fluorinated dendrimer **F0** recorded in CD_3OD at 300K. (**C**) HRMS in positive electrospray mode spectrum recording in acidified methanol

A

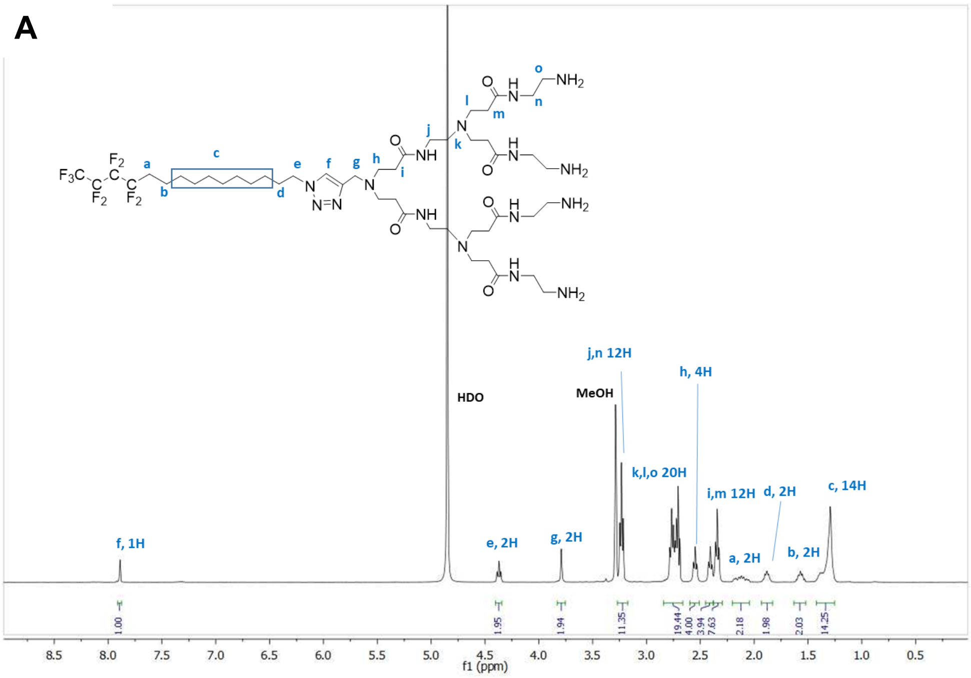

B

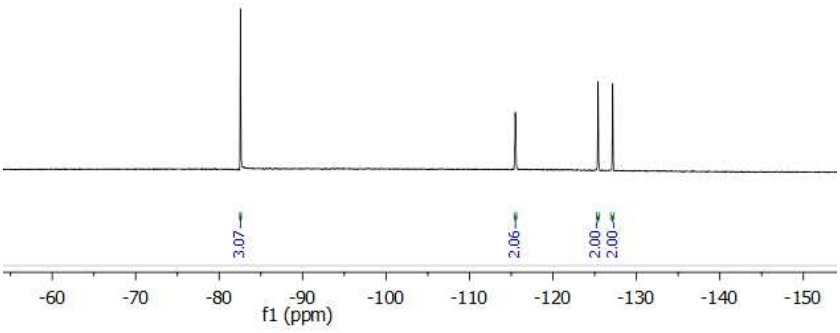

C

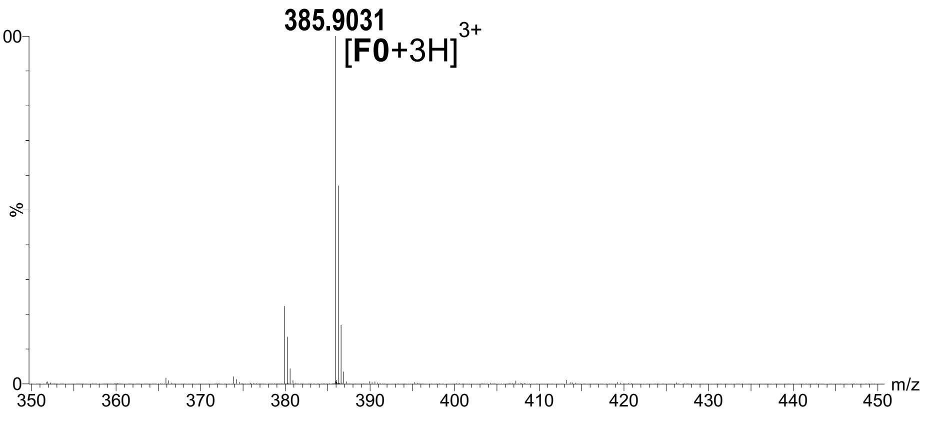

Figure S2: ^{1}H, ^{13}C, ^{19}F, NMR of the fluorinated dendrimer F1

(**A**) ^{1}H NMR 500 MHz (**B**) ^{19}F NMR 470 MHz and -(**C**) ^{13}C NMR 126 MHz spectra of the fluorinated dendrimer **F1** recorded in CD_3OD/D_2O : 1/1 at 300K.

A

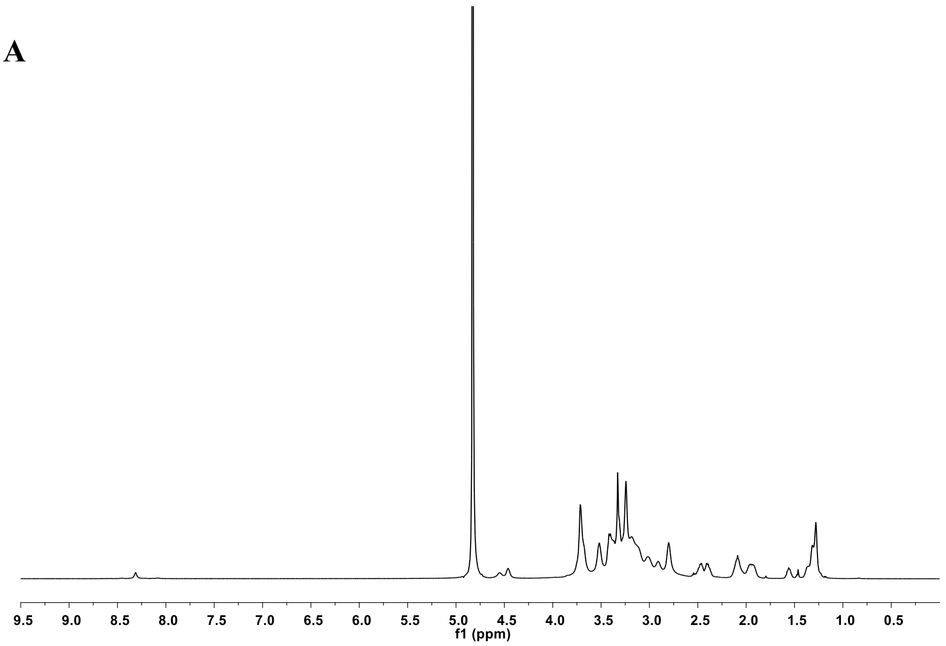

B

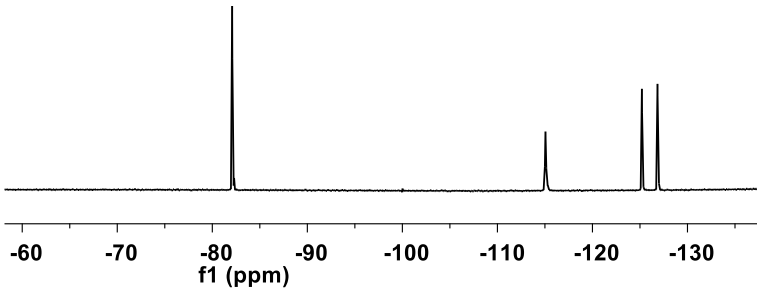

C

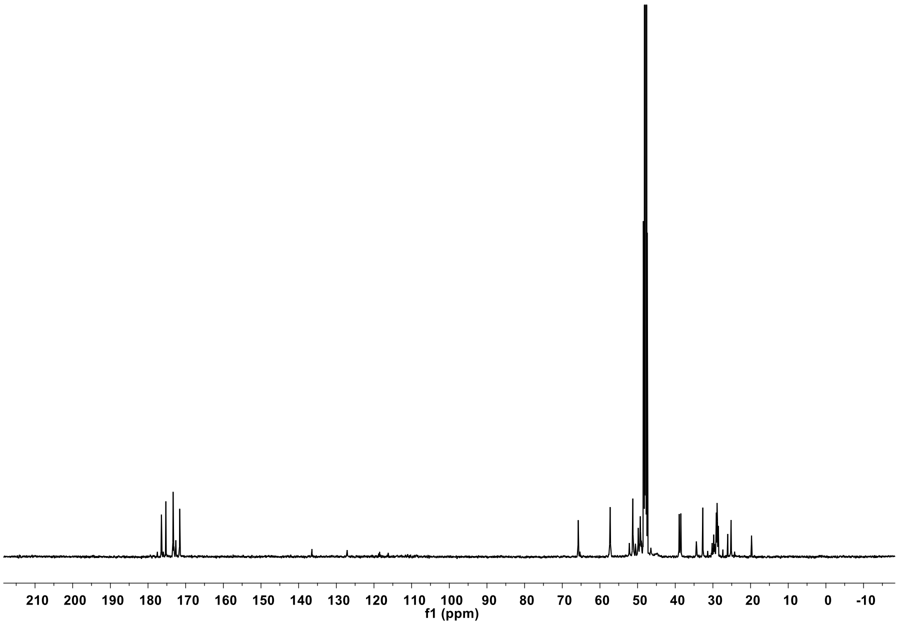

Figure S3: Critical micelle concentrations (CMC) of [^{69}Ga]Ga-F1@

CMC were estimated using fluorescent spectroscopic assay with Nile Red.

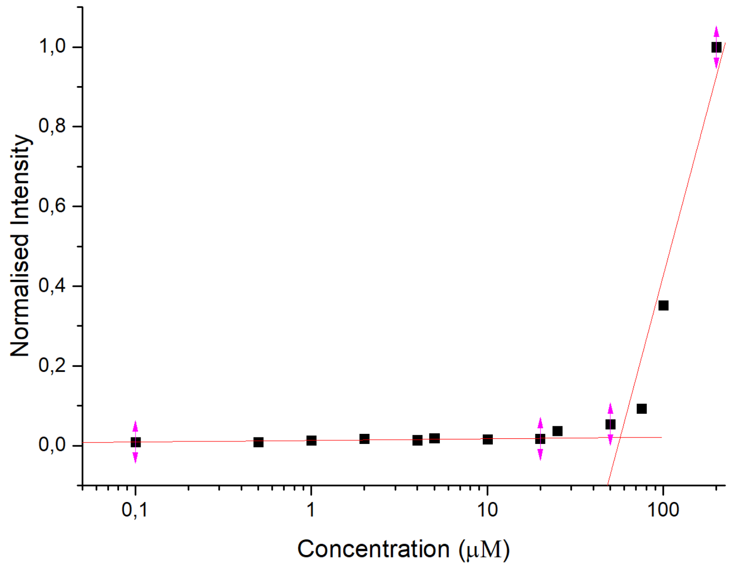

Table S1: Mean radiochemical purity values for [^{68}Ga]Ga-1@ stability

	Serum 37°C	NaCl 37°C	Serum 25°C	NaCl 25°C
0H	97.8±0.1	97.8±0.1	97.8±0.1	97.8±0.1
1H	97.4±0.3	96.9±0.1	97.7±0.1	96.0±0.1
2H	96.5±0.1	96.2±0.3	97.6±0.1	95.7±0.1
3H	96.4±0.3	96.3±0.4	97.2±0.4	95.5±0.3
4H	96.6±0.2	96.4±0.3	96.4±0.8	95.7±0.3

Table S2: Mean radiochemical purity values for [^{68}Ga]Ga-F1@ stability

	Serum 37°C	*NaCl 37°C*	*Serum 25°C*	*NaCl 25°C*
0H	99.0±0.1	99.0±0.1	99.0±0.1	99.0±0.1
1H	97.9±0.1	97.9±0.1	98.3±0.1	97.3±0.1
2H	98.3±0.1	97.7±0.1	97.9±0.1	98.2±0.1
3H	98.3±0.1	98.0±0.1	97.5±0.2	97.8±0.1
4H	98.0±0.1	97.4±0.1	98.3±0.1	97.9±0.1

Table S3 : Mean PET quantifications values for [^{68}Ga]Ga-1@ and [^{68}Ga]Ga-F1@

	ectopic U87 (%ID/cm^3)				orthotopic SOJ6 (%ID/cm^3)			
	[^{68}Ga]Ga-**1@**		[^{68}Ga]Ga-**F1@**		[^{68}Ga]Ga-**1@**		[^{68}Ga]Ga-**F1@**	
Organs	Mean	SD	Mean	SD	Mean	SD	Mean	SD
Bladder	41.94	5.24	106.77	25.06	41.66	21.64	73.46	27.24
Brain	1.84	0.23	1.20	0.16	1.62	0.27	1.07	0.22
Heart	8.27	1.38	4.83	0.87	7.70	1.66	4.47	1.07
Kidneys	8.47	1.60	7.55	0.54	7.97	1.97	9.65	2.99
Liver	7.74	1.46	4.07	0.57	6.15	1.41	3.51	0.76
Lung	3.93	0.71	2.41	0.37	3.79	0.79	2.44	0.55
Muscle	1.75	1.37	0.58	0.60	1.65	0.97	0.88	0.72
